# Matched-Pair Analysis of Survival in the Patients with Advanced Laryngeal and Hypopharyngeal Squamous Cell Carcinoma Treated with Induction Chemotherapy Plus Chemo-Radiation or Total Laryngectomy

**DOI:** 10.3390/cancers13071735

**Published:** 2021-04-06

**Authors:** Patricia García-Cabo, Fernando López, Mario Sánchez-Canteli, Laura Fernández-Vañes, César Álvarez-Marcos, José Luis Llorente, Maria Ángeles de la Rúa, Pilar Blay, Juan P. Rodrigo

**Affiliations:** 1 Department of Otorhinolaryngology, Head and Neck Surgery, Hospital Universitario Central de Asturias (HUCA), 33011 Oviedo, Spain; patty.gcabo@gmail.com (P.G.-C.); mariosanchezcanteli@gmail.com (M.S.-C.); laufva@gmail.com (L.F.-V.); alvarezmarcos@telefonica.net (C.Á.-M.); jllorentep@uniovi.es (J.L.L.); 2 Instituto de Investigación Sanitaria del Principado de Asturias (ISPA), Instituto Universitario de Oncología del Principado de Asturias (IUOPA), University of Oviedo, IUOPA, CIBERONC-ISCIII, 33011 Oviedo, Spain; 3 Department of Radiation Oncology, Hospital Universitario Central de Asturias (HUCA), 33011 Oviedo, Spain; angeles.delarua@sespa.es; 4 Department of Medical Oncology, Hospital Universitario Central de Asturias (HUCA), 33011 Oviedo, Spain; pilar.blay@sespa.es

**Keywords:** organ preservation, laryngectomy, laryngeal cancer, hypopharyngeal cancer

## Abstract

**Simple Summary:**

There are no randomized studies comparing organ-preservation protocols with chemo-selection to the classical total laryngectomy plus (chemo)radiotherapy. Thus, we performed a matched-pair analysis to compare these two treatments with respect to locoregional control, disease-specific survival (DSS), and overall survival (OS) in patients with locally advanced squamous cell carcinoma of the larynx and hypopharynx. The results did not show differences between the two treatments for patients with T3/T4a larynx and T2–T4a hypopharynx cancer with respect to OS and DSS, locoregional control, and metastasis-free survival.

**Abstract:**

Background: We performed a comparative analysis between an organ-preservation protocol and surgery followed by radiotherapy in patients with locally advanced squamous cell carcinoma of the larynx and hypopharynx; Methods: 60 previously untreated patients who were treated with induction chemotherapy followed by chemoradiotherapy in responders were compared with a control group of 60 patients treated with up-front surgery. Both groups were statistically comparable, according to the subsite, TNM (tumor-node-metastasis) stage, age, and sex; Results: Mean age was 58 years and 92% were male. No significant statistical difference was observed for overall survival (OS) (HR 0.75; 95% CI 0.48–1.18; *P* = 0.22) and disease-specific survival (DSS) (HR 0.98; 95% CI 0.52–1.83, *P* = 0.96). Also, there was no significant difference for recurrence-free survival (HR 0.931; 95% CI 0.57–1.71; *P* = 0.81), metastases-free survival (HR 2.23; 95% CI 0.67–7.41; *P* = 0.19), and the appearance of second primary tumors (HR 1.22; 95% CI 0.51–2.88; *P* = 0.64); Conclusions: The results of the organ-preservation approach did not appear inferior to those of surgery plus (chemo)radiotherapy for patients with T3/T4a larynx and T2–T4a hypopharynx cancer with respect to OS and DSS, locoregional control and metastases-free survival.

## 1. Introduction

More than 60% of patients with head and neck squamous cell carcinoma (HNSCC) present with stage III or IV disease. Management of these patients includes definitive non-surgical treatment or up-front surgery followed by adjuvant (chemo)radiation dictated by pathologic risk factors [1].

The larynx performs many essential functions including breathing, speaking, and swallowing. For this reason, the quality of life is significantly affected by laryngeal and hypopharyngeal cancer and its treatment. Therefore, the focus of management for the last few decades has been on preserving the function of the larynx without compromising survival [2,3]. A functional larynx inpatient with locally advanced (T3/T4a) laryngeal or hypopharyngeal cancer is defined as those who have an understandable voice, can swallow without aspiration, and breath normally without the need for tracheostomy [3].

In the early 1980s, the standard treatment for locally advanced laryngeal and hypopharyngeal cancer was total laryngectomy [4,5]. However, this practice changed after some landmark studies published in 1991, 1996, and 2003 that investigated larynx-preserving treatments in locally advanced laryngeal and hypopharyngeal cancers [5,6,7]. The results of these trials showed that non-surgical treatment did not lead to a reduction in survival, allowing control of the disease with preservation of laryngeal function in up to two-thirds of cases. Since then, different strategies have been proposed in an attempt to improve the possibilities of preservation and minimize toxicity in patients with locally advanced laryngeal and hypopharyngeal tumors. Current treatment options to preserve the larynx and its function include primary concurrent chemo-radiotherapy (CRT) or induction chemotherapy (ICT) followed by (chemo)radiotherapy (ICT + RT) [8]. Induction chemotherapy was investigated in order to improve definitive non-surgical management. Urba et al. [9] designed a phase II trial to select patients with advanced laryngeal cancer for nonsurgical treatment. Patients who achieved a partial tumor response to a single test cycle of neoadjuvant therapy (cisplatin 100 mg/m^2^ plus 5-fluorouracil 1000 mg/m^2^/day, 5 days) were selected to definitive chemo-radiotherapy; non-responders underwent immediate surgery and postoperative radiation. This trial reported that one cycle of neoadjuvant chemotherapy can identify laryngeal cancer patients who are highly likely to be successfully treated with CRT. The value of chemotherapy as a screening agent for selecting those patients who will respond to CRT has been noted by other authors [10,11]. However, although ICT could serve to select patients to definitive CRT, it does not offer a survival advantage: in the DeCIDE and PARADIGM trials patients were randomized to CRT alone or ICT followed by CRT, and no significant difference in overall survival (OS) was found [12,13].

There are no randomized studies comparing organ-preservation protocols with chemo-selection to the classical total laryngectomy plus (chemo)radiotherapy. Thus, as an alternative, we performed a matched-pair analysis to compare these two treatments with respect to locoregional control, disease-specific survival, and overall survival in patients with locally advanced squamous cell carcinoma of the larynx and hypopharynx.

## 2. Materials and Methods

### 2.1. Patients

A total of 120 patients with T3/T4a laryngeal and T2–T4a hypopharyngeal squamous cell carcinoma (SCC) treated at the Hospital Universitario Central de Asturias between 1999 and 2016 were reviewed for this retrospective study. The study was approved by the institutional review board of our institution. Given the retrospective and observational characteristics of the study, informed consent for study inclusion was not necessary.

We selected 2 homogeneous groups of patients: 60 previously untreated patients with SCC of the larynx and hypopharynx, who were included between 2007 and 2016 in an organ-preservation protocol consisting in a single cycle of ICT followed by definitive CRT in responders and surgery (plus adjuvant treatment) in non-responders, and a control group of 60 patients treated with up-front surgery between 1999 and 2007. Both groups were statistically comparable, according to disease stage (using the TNM system of the Union for International Cancer Control, 7th Edition), age, and sex. None of our patients had distant metastases at the time of diagnosis. Follow-up was available in all patients for at least 2 years or until death.

From 2007, non-surgical treatment was offered to patients with pathologically confirmed, resectable, previously untreated locally advanced laryngeal (T3 and selected T4a) and hypopharyngeal tumors (T2 to selected T4a) who were candidates for a total laryngectomy. Staging included flexible laryngoscopy and contrast-enhanced computed tomography (CE-CT). Gross invasion of the thyroid or cricoid cartilage or the extra laryngeal soft tissues and the presence of a non-functional larynx was considered a criterion for exclusion. Moreover, patients had to be under 70 years old, they were required to have a Karnofsky performance score of >60% and adequate medical (nutritional status, pulmonary and heart functions) and laboratory status (creatinine clearance >60 mL/min; leukocytes >4000/mm^3^; platelets >100,000/mm^3^) to undergo chemotherapy.

ICT consisted of one cycle of cisplatin, 100 mg/m^2^ administered on day 1, and 5-Fluorouracil (1000 mg/m^2^/day) administered as a 24-h, continuous infusion, for 5 days. All patients were examined by CE-CT 3 weeks after ICT treatment to measure the percentage reduction in the primary tumor. Tumor response was defined by a decrease of at least 50% in the largest tumor dimension according to RECIST (Response Evaluation Criteria In Solid Tumors) criteria [14]. Responders underwent definitive CRT and non-responders underwent surgery followed by (chemo)radiotherapy if indicated. Definitive intensity-modulated RT (IMRT) was administered within 4 weeks after ICT. Treatment was administered once daily, 5 days per week, at 2 Gy per fraction. Large-field treatment included the primary tumor, which involved lymph nodes and nodes at risk. The dose to the initial large fields was 46 to 50 Gy and the dose to the tumor and involved lymph nodes was 70 Gy. Patients received cisplatin 75 mg/m^2^ on days 1, 22, and 43, concurrent with radiation therapy. Twelve weeks after the completion of chemo-radiotherapy, tumor evaluation was performed and recorded separately for the primary tumor and regional nodes. PET-CT scan of the neck and flexible laryngoscopy was performed, with biopsy of any suspected persistent primary lesion. Patients who had residual disease at the neck were eligible for modified radical neck dissection and patients with biopsy-proven disease at the primary site underwent laryngectomy.

Matched-pair controls (according to age, gender, T-classification, and N-classification) were obtained from patients with laryngeal (T3–T4a) and hypopharyngeal (T2–T4a) carcinomas who were treated with up-front total laryngectomy between 1999 and 2007. The indication for total laryngectomy included patients in an appropriate medical condition to tolerate the procedure who were not candidates for organ preservation surgery. None of the patients had distant metastases at the time of diagnosis. Unilateral or bilateral selective neck dissections were performed in conjunction with total laryngectomy for any patient initially staged N0. Ipsilateral or bilateral modified or radical neck dissection was required for any patient initially staged N+. Subsequently, the indications for performing postoperative RT were locally advanced primary tumors (T4), pN2b–N3 cases, and if there was an extranodal extension or if surgical margins were positive. The patients were treated with RT with conventional fractionation (2.0 Gy per fraction, 5 fractions per week). The dose to the tumor bed and lymph nodes was 56 to 64 Gy, depending on extranodal extension or close surgical margins. Patients with positive resection margins received a total of 66 to 70 Gy to the sites of residual disease. 

### 2.2. Statistical Analysis

IBM-SPSS version 19.0 was the statistical software used. The Chi-squared test was used for comparison between the qualitative variables. Survival was calculated using the Kaplan–Meier method, and the differences between survivals were calculated using the logarithmic ranges method. The minimum follow-up of the patients was 24 months or until their death. Cox’s regression model was used for the multivariate analysis. 

Matching was accounted for in the Cox proportional hazard models by incorporating a matching variable that accounted for the matching according to age, gender, nodal classification, and T-classification.

All the tests were bivariate and the level of significance was set at *P* < 0.05.

## 3. Results

### 3.1. Patient Characteristics

Table 1 shows the patient and tumor characteristics. The mean age was 58 years (range, 36 to 73 years; median age, 58 years) and males were predominant (92%). In addition to the matched variables (age, gender, T-classification, and N-classification) there was no significant difference between the two groups for location, histological grade, and AJCC (American Joint Committee on Cancer) stage (*P* > 0.05).

The mean follow-up time was 62 months (range, 1 to 219 months, median follow-up, 49 months).

### 3.2. Organ Preservation Group

All 60 patients in the organ preservation group received one cycle of ICT. Out of 60 patients, 29 (48%) had more than 50% response of the primary tumor site and proceed to definitive RT. In addition, 20 patients received 3 cycles of cisplatin, 6 patients received 2 cycles, 1 patient received 1 cycle, and 2 patients did not receive chemotherapy. Thus, 70% of patients received 3 cycles of concurrent chemotherapy and 90% of patients received at least 2 cycles.

Surgery was proposed for 30 (50%) patients, who were considered as non-responders. In 5 patients a larynx-preservation surgery could be performed: 2 supraglottic laryngectomies, 2 supracricoid laryngectomies, and 1 frontolateral laryngectomy. The remaining 25 patients were treated by total laryngectomy. Of the 30 patients, 14 received postoperative RT (47%); 12 patients had stage IV tumors (86%), and 2 patients had stage III tumors (14%). One patient (2%) died after the cycle of ICT secondary to neutropenia.

Depending on the location, the rate of patients that responded to ICT was 43% in patients with laryngeal tumors (14 patients) and 53% in hypopharyngeal carcinomas (15 patients), meaning that the differences were not significant (*P* = 0.32). Neither significant differences were found in the response to ICT depending on the T and N classification (*P* = 0.9 and *P* = 0.8, respectively). Among patients who received definitive CRT, six (21%) of 29 patients presented locoregional recurrence, two (7%) distant metastases, and seven (24%) developed a second primary tumor. The most frequent location was the lung (4 patients), followed by the head and neck area (2 patients with carcinoma of the tongue) and sigma (one patient).

Patients with locoregional recurrence were surgically salvaged by laryngectomy; therefore, the final laryngeal preservation rate was 79% in the patients that received definitive CRT (39% of total patients treated with ICT).

During the follow-up, five (17%) of 30 patients of the non-responder group presented locoregional recurrence, nine (30%) distant metastases, and three (10%) developed a second primary tumor (2 esophageal carcinomas and one lung carcinoma). 

Treatment-related toxicities were reported in accordance with the Radiation Therapy Oncology Group (RTOG) and the European Organization for Research and Treatment of Cancer (EORTC)scale [15]. After the ICT, 49 patients (82%) experienced some type of toxicity (Table 2). During CRT, 25 of 29 patients (86%) experienced grade 2 or 3 mucositis and 12 of 29 patients (41%) experienced grade 1 or 2 radiodermatitis (Table 2). Only one patient at the end of CRT required a tracheotomy because of laryngeal toxicity, and no patient required a permanent feeding tube.

### 3.3. Surgery Group

All the patients in this group received a total laryngectomy. Three patients did not undergo neck dissection, 10 patients unilateral selective neck dissection (levels II–IV), 37 patients underwent bilateral selective neck dissection, 4 cases underwent radical ipsilateral neck dissection, and 6 patients underwent a combination of selective and radical neck dissections. 25 patients (42%) received postoperative RT: 21 patients (84%) had stage IV disease and 4 patients (16%) had stage III tumors. During the follow-up, seven (12%) of 60 patients presented locoregional recurrence, 12 (20%) distant metastases, and 13 (22%) patients a second primary tumor. The most frequent location was the lung (8 patients), followed by the head and neck area (2 patients with carcinoma of the tongue and one case of the tonsil) and sigma (2 cases).

One patient required a permanent feeding tube due to pharyngoesophageal stenosis secondary to treatment.

### 3.4. Survival Analysis

**Figure 1:**
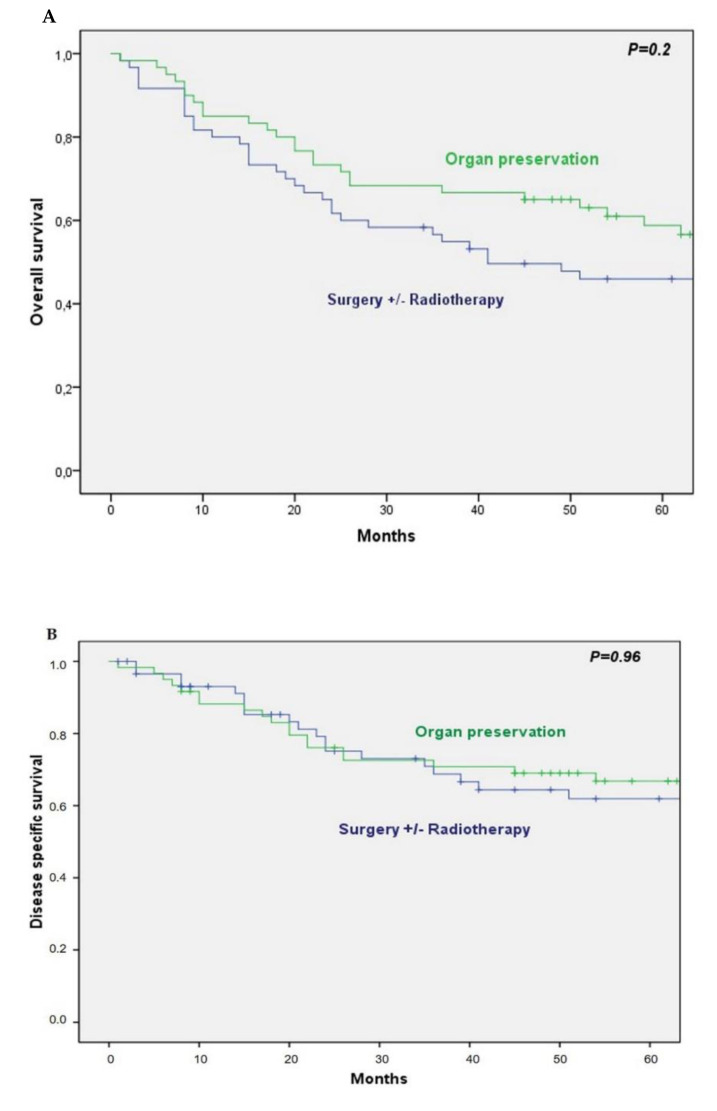
(**A**) Overall survival by treatment; (**B**) disease-specific survival by treatment.

The 5-year overall survival (OS) and disease-specific survival (DSS) in the group of patients undergoing ICT was 59% and 67%, respectively. Five-year OS and DSS in the up-front surgical group was 46% and 62%, respectively. Cox univariate analysis did not show a significant difference in OS (HR 0.75; 95% CI 0.48–1.18; *P* = 0.22) and DSS (HR 0.98; 95% CI 0.52–1.83, *P* = 0.96) between both groups (Figure 1).

**Figure 2:**
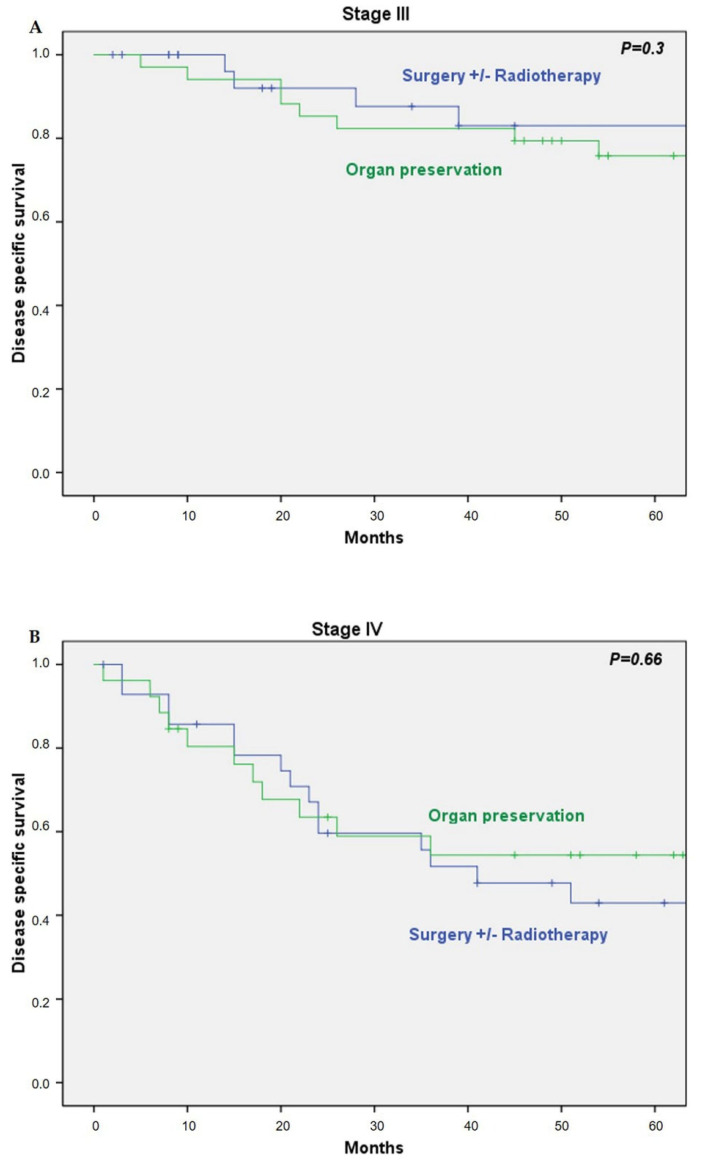
(**A**) Disease-specific survival by treatment in stage III; (**B**) disease-specific survival by treatment in stage IV.

There was no significant difference in DSS between both groups stratified by disease stage (HR 1.81; 95% CI 0.56–5.78; *P* = 0.31 for stage III disease, and HR 0.84; 95% CI 0.38–1.8; *P* = 0.66 for stage IV disease) (Figure 2).

**Figure 3:**
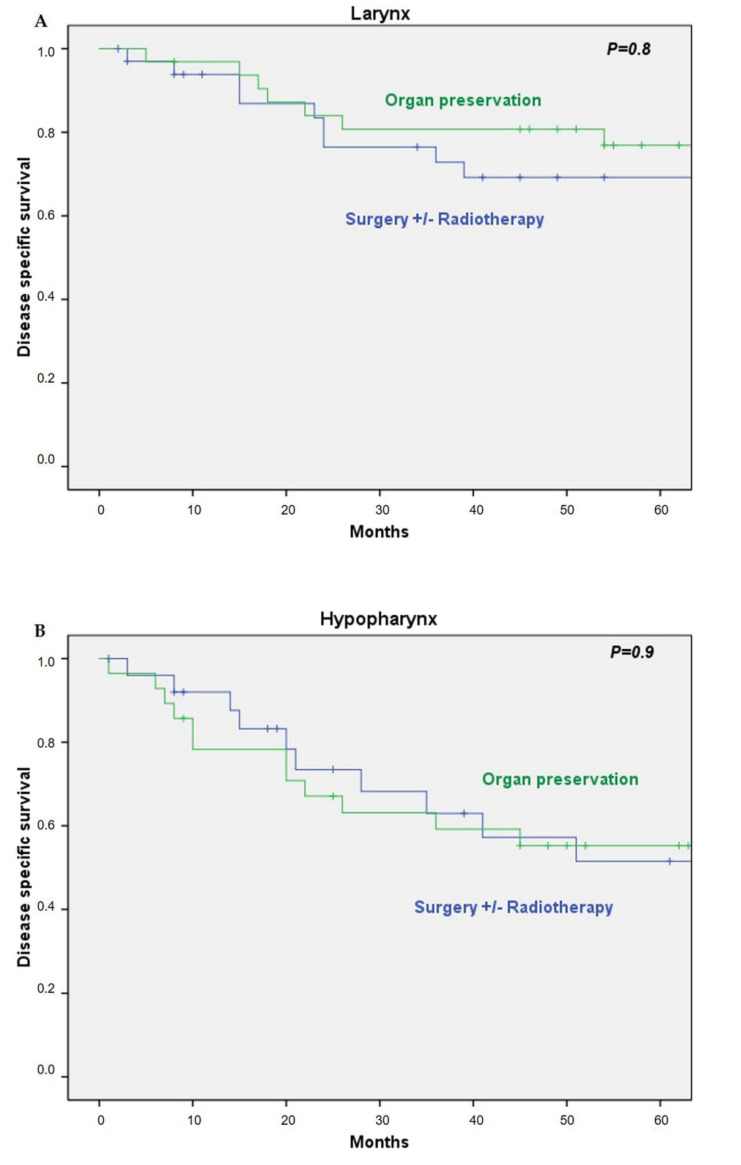
(**A**) Disease-specific survival by treatment in the larynx; (**B**) disease-specific survival by treatment in the hypopharynx.

There was also no difference in DSS between the two treatment groups as a function of tumor localization. The 5-year DSS in the larynx was 77% in the ICT group and 69% in the up-front surgical group (HR 0.9; 95% CI 0.36–2.28, *P* = 0.83). In the hypopharynx, the 5-year DSS was 55% and 52% in the ICT and surgical groups, respectively (HR 1.03; 95% CI 0.44–3.39, *P* = 0.94) (Figure 3).

**Figure 4:**
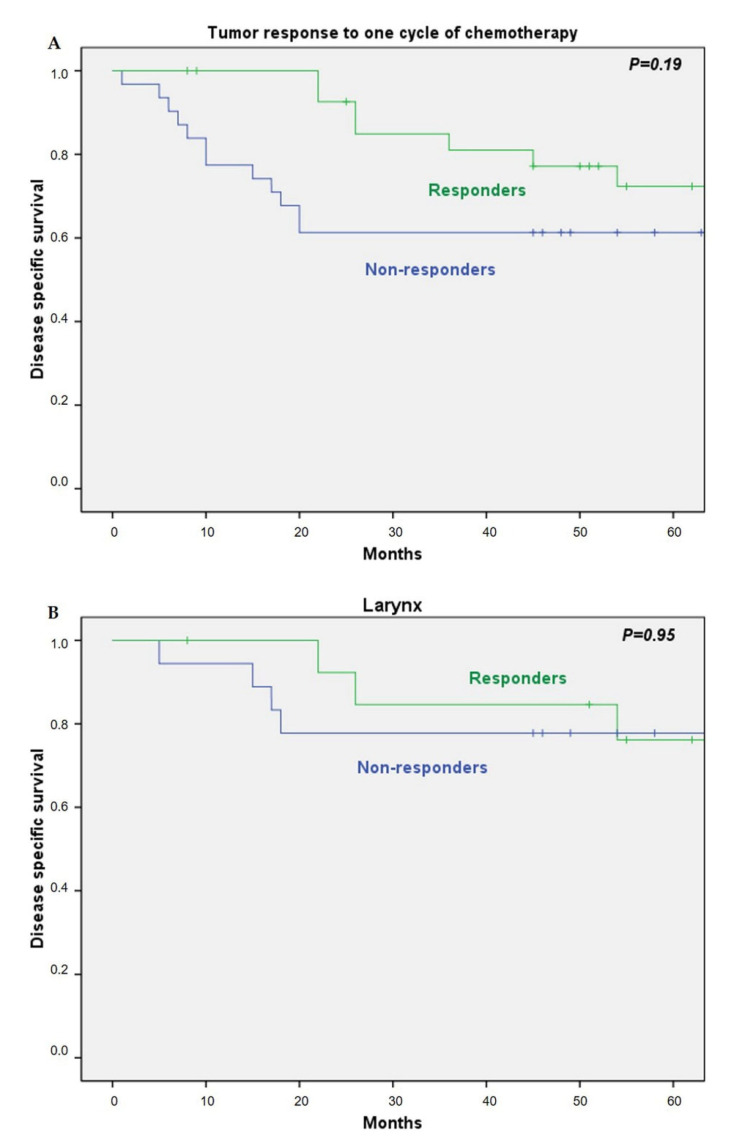

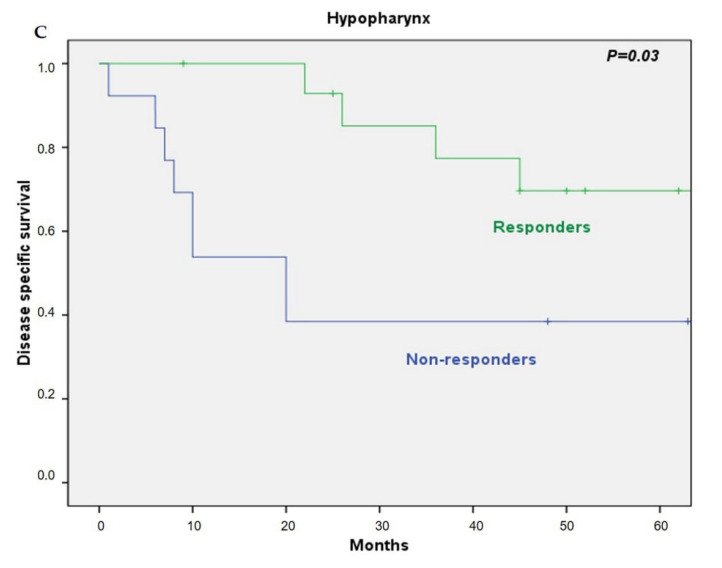
Disease-specific survival dependent on the response after one cycle of chemotherapy: (**A**) all patients; (**B**) tumor location in larynx; (**C**) tumor location in the hypopharynx.

In the group of patients that received ICT, there were no significant differences in the 5-year DSS in responders and non-responders (72% vs. 61%, respectively, *P* = 0.19; Figure 4A). However, when we analyzed the DSS in responders and non-responders by tumor location, we observed that in laryngeal tumors the response to ICT did not significantly influence the 5-year DSS (76% in responders vs. 78% in non-responders, HR 1.04; 95% CI 0.27–3.9; *P* = 0.95; Figure 4B), but in hypopharyngeal tumors, the 5-year DSS was significantly lower in the non-responders (70% in responders vs. 38% in non-responders, HR 3.54; 95% CI 1.05–11.88; *P* = 0.03; Figure 4C).

**Figure 5:**
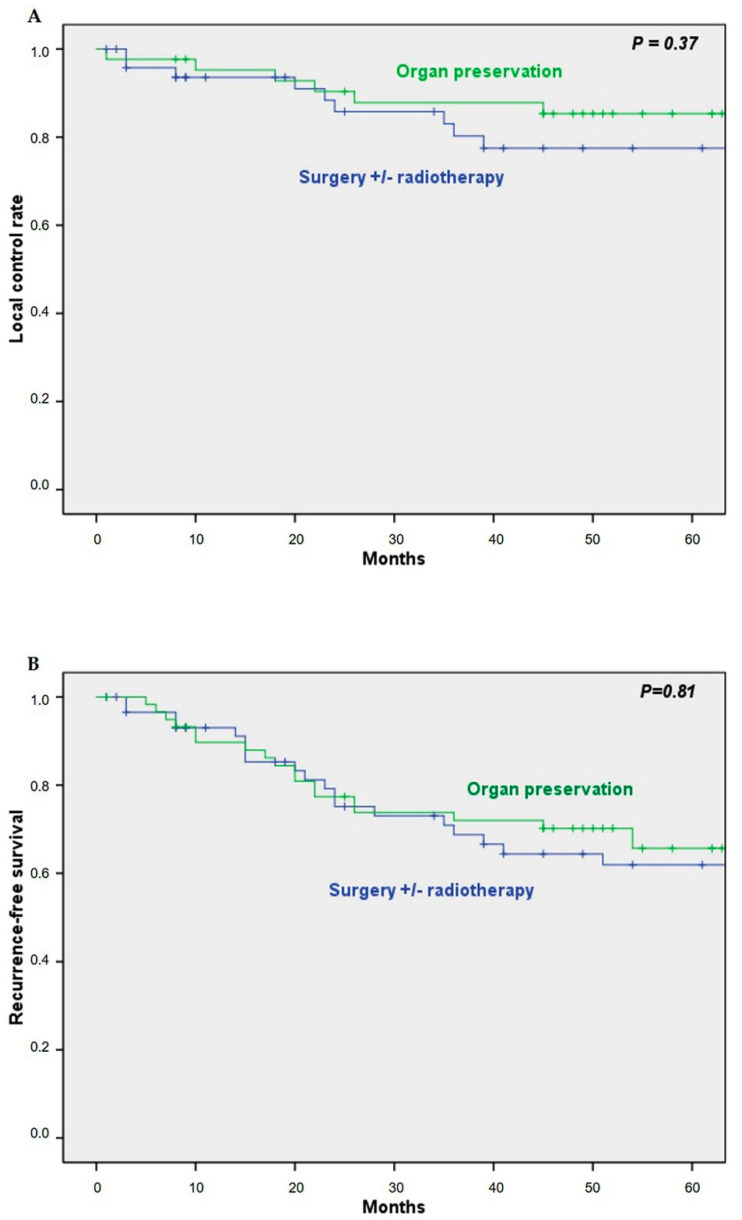

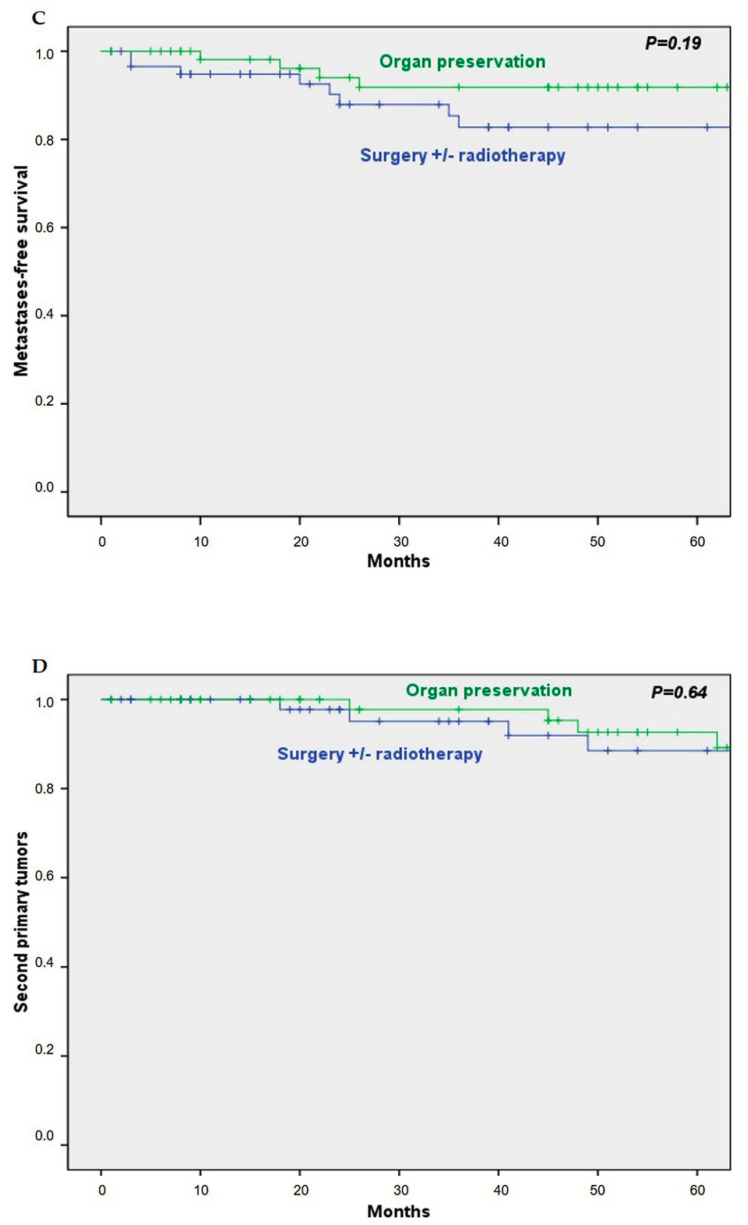
Kaplan-Meier curves for local control rate: (**A**) recurrence-free survival; (**B**) metastasis-free survival; (**C**) second primary tumors-free survival; (**D**) among patients treated with the organ preservation protocol or with up-front surgery.

Similar to the OS and DSS, there were no significant differences between the ICT group and the up-front surgery group for local control rate (HR 0.63; 95% CI 0.22–1.77; *P* = 0.37), recurrence-free survival (HR 0.931; 95% CI 0.57–1.71; *P* = 0.81), metastases-free survival (HR 2.23; 95% CI 0.67–7.41; *P* = 0.19) and the appearance of second primary tumors (HR 1.22; 95% CI 0.51–2.88; *P* = 0.64) (Figure 5).

Multivariable logistic regression analysis included sex, age, alcohol, and tobacco use, T- and N- classification, disease stage, and histological grade. Only the presence of nodal metastasis had a significant impact on survival outcome (OR 1.84; 95% CI 1.25–2.7; *P* = 0.02).

### 3.5. Matched Analysis of Survival

Compared with primary surgical treatment, ICT was not associated with an increased risk of death and progression (Table 3).

## 4. Discussion

In locally advanced laryngeal and hypopharyngeal carcinoma different treatment strategies combining radiotherapy and chemotherapy have been proposed as an alternative to up-front surgery, which usually implies a total laryngectomy, with the aim to preserve the laryngeal function. But any alternative option only is acceptable if it provided similar treatment results as surgery plus radio(chemo)therapy. Our results show that a larynx preservation protocol for advanced laryngeal and hypopharyngeal cancer based on a selection of patients with ICT seemed safe and efficient in terms of loco-regional control, metastases-free survival, DSS, and OS.

The standard treatment for locally advanced laryngeal and hypopharyngeal cancer was total laryngectomy up to Veterans Affairs (VA) larynx trial [5] in 1991 and the EORTC trial [6] in 1996. Subsequently, the RTOG 91-11 study in 2003 demonstrated the superiority of concurrent CRT over ICT plus RT and RT alone in terms of laryngeal preservation [7]. In the VA study [5], the 3-year OS and DSS were 69% and 76%, for patients treated with the organ preservation protocol and surgery, respectively. At 3 years, the survival rate in the EORTC trial [6] seemed superior for the chemotherapy-arm patients (57%) than for patients entered in the surgery arm (43%); in contrast, at 5 years there was no difference between the two treatment arms (30% in the chemotherapy arm versus 35% in the surgery arm). The long-term results of the RTOG 91-11 [7,16] study showed that locoregional control and larynx preservation were significantly improved with concomitant cisplatin/RT compared with the induction arm or RT alone (81.7%, 67.5%, and 63.8% in the concomitant, induction, and RT alone arms, respectively), but 5-year OS did not differ significantly between the three arms (58% for induction, 55% for concomitant, and 54% for RT alone).

Matched-pair analysis has been used in several retrospective cohort studies of head and neck cancer [17,18,19,20], however, none has compared chemo-selection with up-front surgery in laryngeal and hypopharyngeal cancer in clinical practice. Therefore, to assess the safety and efficacy of a larynx-preservation treatment protocol based on chemo-selection with one cycle of ICT, we selected two homogeneous groups of patients matched for age, sex, location, and disease stage treated either with the larynx-preservation protocol or by up-front surgery.

Our results showed non-significant differences in 5-year OS and DSS between the chemo-selection treatment group (59% and 67%, respectively) and the surgical group (46% and 62%, respectively). These results compare well with the 5-year OS rates reported in randomized trials [5,6,7,16] and with other retrospective studies that reported 5-year OS rates between 36% and 58% [21,22,23]. Like our study, Rades et al. [17] conducted a paired study for nine potential prognosis factors: they presented 44 patients treated with definitive CRT matched (1:2) to 88 patients treated with surgery plus CRT. The treatment regimens did not significantly differ with respect to loco-regional control, metastases-free survival, and overall survival (67% and 63% for surgery plus CRT and CRT, respectively).

Since the VA study and subsequent trials, different strategies have been proposed in an attempt to improve the possibilities of laryngeal preservation and minimize toxicity in patients with locally advanced laryngeal and hypopharyngeal tumors. The value of chemotherapy as a screening agent for selecting those patients who will respond to CRT has been noted by several authors [5,7,10,11,24]. Urba et al. [9] demonstrated that one cycle of induction chemotherapy (cisplatin and 5-fluorouracil) identifies a group of patients whose laryngeal cancer is highly likely to be successfully treated with definitive CRT, achieving a 70% larynx preservation rate and excellent function. Their estimated DSS and OS rates at 3 years were 87% and 78%, respectively. Our larynx preservation results (39%) are substantially lower, mainly due to a lower response rate to ICT, despite the use of the same ICT scheme. Urba et al. [9] reported a 75% response rate to ICT in contrast with our 48% response rate. Moreover, differences in the local persistence/recurrence rates after definitive CRT in responders to ICT were smaller: 12% in the series of Urba et al. [9] and 21% in our series. The inclusion of patients with hypopharyngeal tumors in our series could be responsible for these differences. Other studies using a single cycle of ICT also showed higher response rates than our study, although the ICT schemes, response criteria, and the characteristics of the patients were different [25,26]. Semrau et al. [25] reported a series of 62 patients with laryngeal, oropharyngeal, and hypopharyngeal cancers showing a 77% response rate to one cycle of cisplatin and docetaxel, with a 13% local recurrence rate in the patients that received definitive CRT, and Wolf et al. [26] reported an 84% response rate to one cycle of cisplatin and fluorouracil, with a 22% local recurrence rate in their series of advanced laryngeal cancers. Interestingly, the study of Wolf et al. [26] showed superior survival rates with the chemo-selection treatment approach using a single cycle of neoadjuvant chemotherapy than with concurrent CRT, comparable to the survival rates achieved in patients selected for primary surgery. Although our laryngeal preservation rates were inferior, our results also support the use of the chemo-selection approach in terms of efficacy since this approach did not significantly differ from primary surgery with respect to OS and DSS.

Our results are consistent with historical evidence [6,7,16,21,27,28] that responders to ICT have a survival advantage compared with non-responders, but in our series, the differences were only statistically significant in hypopharyngeal cancers. Survival outcomes differ in the different subsites of head and neck cancer, with the hypopharyngeal subsite having one of the worst prognoses. This is also observed in our series, where the 5-year DSS in the chemo-selection group was 77% in laryngeal and 55% in hypopharyngeal tumors, and 69% and 52%, respectively, in the primary surgery group.

One of the objectives of the chemo-selection approach with one cycle of ICT is to avoid the toxic effects of CRT in the patients that will not benefit from this treatment. Concurrent CRT is associated with a high rate of acute and late toxic effects. The RTOG 91–11 trial [7] reported a total rate of severe toxic effects for all phase of the study of 81% with induction cisplatin plus fluorouracil followed by radiotherapy and 82% with radiotherapy with concurrent cisplatin, vs. 61% with radiotherapy alone, and the total numbers of deaths that may have been related to treatment were 3%, 5%, and 3%, respectively. Due to the toxic effects, only 70% of patients received three cycles of concurrent chemotherapy. Although also more than 80% of our patients who received CRT experienced toxic effects, less toxicity secondary to chemotherapy is observed in possible relation to the lower cisplatin dose administered (75 mg/m^2^). However, only 70% of patients had received three cycles of concurrent chemotherapy, although 90% of patients received at least two cycles. The lower toxicity is also reflected in that only one patient who concluded organ preservation chemoradiotherapy needed a tracheotomy and no patient required a permanent feeding tube.

Other studies using the standard cisplatin dose (100 mg/m^2^) reported higher rates of toxicity related to CRT. Urba et al. [9] reported a 2.7% rate of permanent feeding tube dependence (3 of 73 patients) with 60% of patients that had received the 3 cycles of concurrent chemotherapy. Timme et al. [29] reported a 38% rate of laryngeal dysfunction at 2 years and an 18% rate of long-term feeding tube dependence. Therefore, it seems that our modality of concurrent CRT with a lower cisplatin dose reduces toxicity without a decrease in treatment efficacy compared to previous studies [9,23,29,30].

Matched-pair analysis has some intrinsic limitations; although the patients in this study were matched for age, sex, T- and N-stage, there are still variables that can bias potentially prognostic factors, such as subsites or arytenoid fixation in relation to functional outcome. Another limitation of our study is that it is retrospective and there might be a selection bias. Matched-pair analysis cannot replace prospective cohort studies, and randomized controlled trials are required for proper comparisons between therapeutic strategies, but in absence of these studies, it is one of the best alternatives.

## 5. Conclusions

In summary, the results of our organ preservation approach did not appear inferior to those of surgery plus radio(chemo)therapy for patients with T3/T4a larynx and T2–T4a hypopharynx cancer with respect to overall and specific disease survival, locoregional control, and metastases-free survival.

One cycle of induction chemotherapy is able to identify laryngeal and hypopharyngeal advanced carcinomas who are likely to be successfully treated with chemoradiotherapy.

## Figures and Tables

**Table 1:** Patient characteristics.

Characteristics	All Patients *N* = 120 (%)	Surgery Group *N* = 60 (%)	Organ Preservation Group *N* = 60 (%)	*P* ^1^
Age				
<55 years	38 (32)	19 (32)	19 (32)	Matched
55–59 years	28 (23)	14 (23)	14 (23)	
>59 years	54 (45)	27 (45)	27 (45)	
Gender				
Female	10 (8)	5 (8)	5 (8)	Matched
Male	110 (92)	55 (92)	55 (92)	
Alcohol				
No-light drinker	56 (46)	34 (56)	25 (41)	0.14
Moderate-heavy drinker	64 (54)	26 (44)	35 (59)	
Tobacco				
No-light smoker	56 (46)	41 (68)	15 (25)	0.1
Moderate-heavy smoker	64 (54)	19 (32)	45 (75)	
Location				
Hypopharynx	54 (45)	26 (43)	28 (47)	
Larynx	66 (55)	34 (57)	32 (53)	0.71
Glottis	21 (32)	12 (35)	9 (28)	
Supraglottis	45 (68)	22 (65)	23 (72)	0.68
T classification				
2	4 (3)	2 (3)	2 (3)	Matched
3	102 (85)	54 (85)	54 (85)	
4	14 (12)	7 (12)	7 (12)	
N classification				
N0	52 (43)	26 (43)	26 (43)	Matched
N1	16 (13)	8 (13)	8 (13)	
N2	48 (40)	24 (40)	24 (40)	
N3	4 (3)	2 (3)	2 (3)	
Disease stage				
III	65 (54)	31 (52)	34 (57)	0.9
IV	55 (46)	29 (48)	26 (43)	
Histological grade				
G1	43 (36)	22 (37)	21 (35)	0.9
G2	46 (38)	22 (37)	24 (40)	
G3	31 (26)	16 (26)	15 (25)	
Follow-up mean (median), months	62 (49)	61 (40)	63 (54)	0.41

^1^ Chi-squared test. *P*-value comparing who received primary surgery vs. patients treated with organ preservation.

**Table 2:** Chemotherapy Toxicities.

Toxicities	Induction Chemotherapy *N* = 60 (%)	Concurrent Chemoradiation *N* = 29 (%)
No toxicity	11 (18)	4 (14)
Mucositis	11 (13)	25 (86)
Neutropenia	3 (5)	4 (14)
Diarrhea and sickness	6 (10)	12 (41)
Renal tubulopathy	8 (13)	5 (17)
Radiodermatitis	0	12 (41)
Other *	1	0

* One sudden death after one cycle of ICT (induction chemotherapy).

**Table 3:** Hazard ratios for event rate associated with ICT.

Cox’s Regression Analysis on Matching Variables	Value
Overall Survival	
Hazard ratio	0.758
95% Confidence interval	0.48–1.18
*P*	0.22
**Disease-Specific Survival**	
Hazard ratio	0.984
95% Confidence interval	0.52–1.83
*P*	0.96

## Data Availability

The data presented in this study are under the custody of the Health Service of the Principality of Asturias and are therefore unavailable for sharing. The data are available on request from the corresponding author after an appropriate data sharing and access agreement is formally completed.
